# Cognitive impairment in children with 5q-associated spinal muscular atrophy type 1: two case reports and the review of the literature

**DOI:** 10.3389/fped.2024.1407341

**Published:** 2024-09-27

**Authors:** Hua Yang, Jie Yang, Yawen Xue, Lihui Liao, Qianyun Cai, Rong Luo

**Affiliations:** ^1^ Department of Pediatrics, West China Second University Hospital, Sichuan University, Chengdu, China; ^2^ Key Laboratory of Obstetric & Gynecologic and Pediatric Diseases and Birth Defects of Ministry of Education, Sichuan University, Chengdu, China; ^3^ Department of Pediatric Neurology Nursing, West China Second University Hospital, Sichuan University, Chengdu, China

**Keywords:** spinal muscular atrophy type 1, motor neuron disease, cognitive impairment, children, case report

## Abstract

Spinal muscular atrophy (SMA) is an autosomal recessive disease caused by mutations in the survival motor neuron 1 (SMN1) gene on chromosome 5, leading to the degeneration of lower motor neurons. There are few studies on cognitive impairment comorbid with SMA. Here, we report two cases of severe cognitive impairment in Chinese children with SMA type 1, marking the first such reports in this demographic. We propose that severe cognitive dysfunction may be a comorbidity of SMA. Clinicians should consider SMA in patients presenting with severe muscle weakness and atrophy accompanied by cognitive impairments, to avoid misdiagnosis and oversight.

## Introduction

Spinal muscular atrophy (SMA) is an autosomal recessive genetic disease affecting motor neurons in the anterior horn caused by survival motor neuron 1 (SMN1) gene (5q11.2) mutation. The main manifestations include progressive muscle weakness and atrophy, primarily in the proximal limbs. Severely affected pediatric patients often succumb to respiratory failure. It has an incidence of approximately 1:10,000 (1, 2). According to the age of onset, motor milestones and the progress of the disease, SMA is divided into 0–4 types. SMA type 0 is defined by prenatal onset and is characterized by limited intrauterine activity. SMA type 1 represents the most serious infant phenotype. Symptoms typically manifest within the first six months after birth, with the maximum motor ability falling short of sitting unassisted. Most children die of respiratory failure within 2 years. SMA type 2 generally presents between 7 and 18 months of age, with patients able to sit independently but unable to stand or walk alone. SMA type 4 starts in adulthood, progresses slowly, and life expectancy is not shortened (3).

Reports often suggest that children with SMA exhibit higher cognitive abilities compared to healthy peers of the same age. Clinicians have noted their keen interest in the environment, sharp mental acuity, and observational skills, despite significant physical limitations (4). The development of cognitive skills may be a creative way to make up for its own limitations (5). For many years, researchers have been focusing on the study of SMA children's motor, breathing, and swallowing system, but cognitive development has not received much attention. SMA (mainly types 1 and 2) is associated with severe weakness, which affects hand coordination and speech acquisition. Limited interaction between speech and sensorimotor skills may lead to cognitive impairments (6, 7).

Cognitive abilities are frequently impacted in progressive neurodegenerative diseases, yet there is limited knowledge regarding cognitive deficits in SMA patients. Due to the severity of the condition, patients with SMA type 0 may also experience significant impacts on cognitive development. Severe neurological involvement is likely to represent the ultimate manifestation of an extreme phenotype of SMA type 0, resulting from a significant reduction of SMN protein levels in the brain (8). There are very few studies on cognitive impairment in SMA type 0. Although some SMA type 1 patients may not show significant cognitive impairments, early motor function damage can indirectly affect cognitive abilities. SMA type 1 patients often exhibit difficulties in language comprehension and memory (5, 9). Cognitive function in SMA type 2 patients is generally better, but they may still display mild language and learning difficulties (10). Research has found that cognitive impairments in this type are relatively few and less severe. For SMA type 3 patients, cognitive impairments are less common, though some individuals may experience minor attention or learning problems (11, 12). SMA Type 4 usually has minimal or no effect on cognitive function, and there are few studies on cognitive impairment in SMA Type 4 (13).

We aim to describe and discuss the factors and pathogenesis affecting cognitive function in SMA through the study of two cases of 5q- SMA children's cognitive dysfunction. We reviewed the literature to assess the cognitive performance of children with SMA and possible differences in cognitive performance among children with different subtypes of SMA (see Tables 1, 2).

**Table 1:** Summary of studies of 5q-SMA with cognitive dysfunction.

Study	Country	SMA type	*N*	Other groups	*N*	Age (years)	Evaluation protocol	Main findings
Billard et al. (14)	France	1, 2	11	DMD/HC	21/42	8.3–13.6	WISC-R; Batterie d’ Evaluation du langage; French language reading test	DMD children exhibited a reading age which was lower than the SMA children compared with their chronological age.
Von Gontard et al. (4)	Germany	1,2,3	96	Non-affected siblings/HC	45/59	6–18.1	CPM/SPM; K-ABC; Wechsler subtests	Children and adolescents with SMA have a general intelligence in the normal range. They develop effective and useful strategies to ‘compensate’ their physical handicap by the acquisition of cognitive skills and knowledge.
Riviere et al. (15)	France	2	12	HC	12	1.5–3	Spatial search skills	The performance obtained with the SMA group did not differ from HC group. Dyskinesia does not appear to be a key risk factor for dramatic slowing down or deviation in the development of spatial search skills.
Polido et al. (7)	Brazil	1	12	HC	12	3–4.5	Pair-matching tasks	Pair-matching performance of SMA-I children was poorer than HC group. The restricted interaction with the environment, due to severe paralysis and poor verbal communication, is associated with cognitive difficulties in SMA-I children.
Osmanovic et al. (16)	Germany	2,3,4	34	ALS	34	19–64	ECAS; WST	SMA patients performed better than ALS patients in the cognitive domains of memory, language and executive function. Better cognitive abilities in SMA patients seemed to be related to the early onset, rather than the extent or the duration, of their physical handicap.
Mix et al. (17)	Germany	2,3	31	HC	19	19.1–64.6	ECAS	No differences between SMA type 2 and SMA type 3 were detected for any domain. A significant inverse correlation of physical function and executive function was detected: lower motor function was associated with a better executive function.
Schmidt et al. (18)	Germany	1,2	8	DMD	8	6–12	WISC-V; AID 3; ZAREKI-R; RNG Task; DCD Task	Children with SMA performed above average in general cognitive and arithmetic tasks. Participants with DMD, revealed spatial-numerical associations comparable to those expected of healthy children but showed weaker performance in arithmetic and in general cognitive ability and executive function.
Kizina et al. (19)	Germany	1,2,3	33	/	/	18–58	WAIS-IV	The IQ index scores for Working Memory and Perceptual Reasoning were lower in the patients with SMA type 2 than the normal population. The IQ index scores of SMA type 3 patients were not different from the normal population, but there was a trend towards lower cognitive performance.
Zappa et al. (20)	Italy	1	22	/	/	0.1–9.0	CPM; TCGB; ALSSS	Despite deprivation of normal developmental stimulation and of very severe limitations in motor function, it appears that intellectual skills and internal language, and particularly morphosyntactic comprehension, are not affected in SMA type 1 children whereas quality of speech is severely affected and dependent on disease severity.
Lenzoni et al. (21)	Italy	3	22	HC	22	18–56	Comprehensive neuropsychological battery	SMA patients showed poorer performance in visuospatial abilities, executive functions and language as compared to healthy controls. In the SMA sample, patients with greater motor difficulties had lower performance in attention, but higher performance in measures of language, verbal fluency, and memory. Cognitive test performance was associated with motor functioning in men.
Buchignani et al. (10)	Italy	2,3	57	/	/	3.5–17	WPPSI-III; WISCIV	Working memory had lower scores in SMA type 3 patients compared to type 2. Intellectual disability is uncommon in type 2 and type 3.
Hu et al. (12)	China	3	22	HC	20	25–34	MoCA; AVLT; DS; VFT; WCST	Patients with SMA type 3 showed selective deficits in executive function.
Steffens et al. (22)	Germany	1	20	/	/	0–2.5	WPSSI-IV; BSID-III	Treated patients with SMA type 1 have heterogeneous cognitive function with 55% of patients showing deficits.

DMD, duchenne muscular dystrophy; SMA, spinal muscular atrophy; CPM, raven’ color progressive matrices; SPM, raven's standard progressive matrices; K-ABC, kaufman assessment battery for children; ALS, amyotrophic lateral sclerosis; ECAS, edinburgh cognitive (and behavioural) ALS screen; WST, German vocabulary test (Wortschatz test); WPPSI-III, wechsler preschool and primary scale of intelligence, ThirdEdition; WISC, wechsler intelligence scale; AID 3, adaptive intelligence diagnosticum 3rd Edition; ZAREKI-R, neuropsychologische testbatterie für zahlenverarbeitung und rechnen bei kindern—revidierte fassung; RNG, random number generation; DCD Task, dot counting direction task; IQ, intelligence quotient; TCGB, test of grammatical comprehension for children (test di comprensione grammaticale per bambini); ALSSS, ALS severity score; MoCA, the montreal cognitive assessment; AVLT, auditory verbal learning task; DS, digital span; VFT, verbal fluency test; WCST, Wisconsin card sorting task; BSID-III, the bayley scales of infant development.

**Table 2:** Overview of cognitive impairments in different types of spinal muscular atrophy (0–4).

SMA type	Description	Cognitive impairments	Severity level	References
SMA 0	Severe early-onset, profound motor impairment	Widespread cognitive impairments, severe language and memory issues	Severe	/
SMA 1	Infantile onset, significant motor limitations	Difficulties in language comprehension and memory	Moderate	Billard et al. (14); Polido et al. (7); Zappa et al. (20); Steffens et al. (22)
SMA 2	Intermediate onset, moderate motor function impairment	Mild language and learning difficulties	Normal/Mild	Riviere et al. (15); Schmidt et al. (18); Kizina et al. (19)
SMA 3	Late-onset, relatively mild motor symptoms	Minor attention or learning issues	Normal/Minimal	Von Gontard et al. (4); Mix et al. (17); Lenzoni et al. (21); Buchignani et al. (10); Hu et al. (12)
SMA 4	Adult-onset, mild motor impairment	Not obvious	None	Osmanovic et al. (16);

## Case presentation

### Case 1

**Figure 1:**
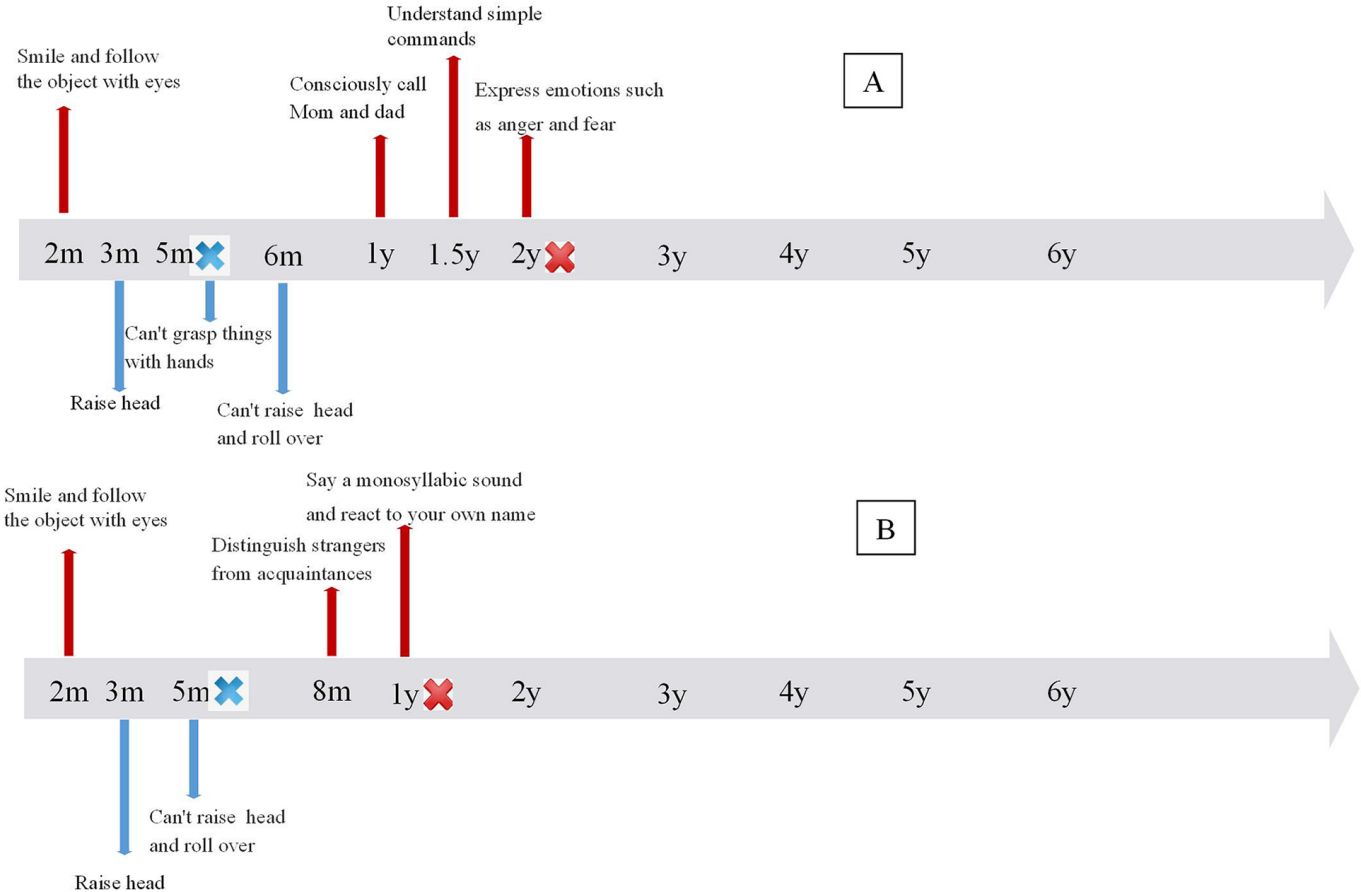
Time line of cognitive and motor development in 2 patients. **(A)** Timeline of Patient 1, **(B)** timeline of Patient 2.

The patient was a 6-year-old girl who first presented to our outpatient clinic at 6 months of age, exhibiting an inability to roll over, sit, or stand independently. She was born at full term, and her birth history was unremarkable. Her parents are healthy and unrelated. She was able to raise her head at 3 months, but at 5 months, she could not grasp objects with her hands. After 6 months, her motor function further deteriorated, and she lost the ability to hold her head up, roll over, or sit independently. She also exhibited cognitive dysfunction. By 12 months, she could consciously say “dad” and “mom” and respond to her name. After 18 months, she could only understand simple instructions. She had memory disorders and could not perform counting or simple calculations (see Figure 1A). Neurological examination revealed bilateral flexion of the knees and toes. Neither knee nor Achilles tendon reflexes could be elicited, and there was a decrease in limb muscle tone and strength. At 4.5 years of age, cognitive functioning was assessed using the Wechsler Preschool and Primary Scale of Intelligence, Fourth Edition, Chinese version (WPPSI-IV). The intelligence quotient (IQ) score was ≤40. The Verbal Comprehension Index, Visuospatial Index, Fluid Reasoning Index, Working Memory Index, and Processing Speed Index were all ≤45, indicating low competence in each subscale.

**Figure 2:**
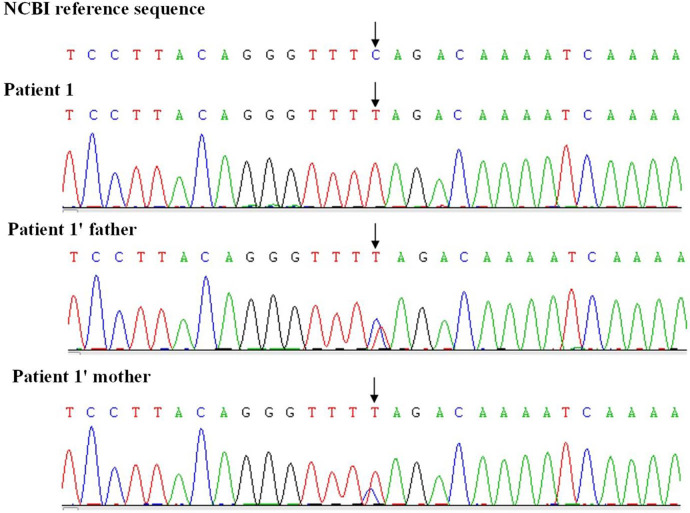
Whole exome sequencing of patient 1.

Results of routine blood tests, blood biochemistry, lactic acid, blood ammonia, and muscle enzyme levels were normal. Brain MRI showed slightly enlarged bilateral lateral ventricles. Needle electromyography revealed chronic denervation. MRI of the leg muscles showed atrophy of the bilateral thigh and buttock muscles. Spinal x-ray revealed scoliosis with a Cobb angle of 33 degrees. Whole exome sequencing identified homozygous mutations in exons 7 and 8 (c.840C>T) of the SMN1 gene on chromosome 5q13 (see Figure 2). The MLPA assay revealed a copy number of 0 for exons 7 and 8 of the SMN1 gene and a copy number of 3 for exons 7 and 8 of the SMN2 gene.

At 5 months of age, the patient initially presented with clinical symptoms indicative of a neuromuscular disorder. Whole exome sequencing and MLPA identified an increased copy number of SMN2, leading to a definitive diagnosis of 5q-associated spinal muscular atrophy type 1. She received four loading treatments and three maintenance treatments of nusinersen from 4.8 years of age. Post-treatment WPPSI-IV testing showed an IQ score of ≤40 and scores <45 on all subtests, suggesting no improvement in cognitive function. The Children's Hospital of Philadelphia Infant Test of Neuromuscular Disorders (CHOP-INTEND) showed no significant improvement in motor function.

### Case 2

The child, an 8-year-old boy, was first admitted to our outpatient clinic at 8 months due to an inability to turn over and sit independently. He was born full term with an unremarkable birth history. His parents are healthy and unrelated. Initially, the patient could hold his head up at 2–3 months, but was unable to hold his head up and roll over after 5 months and unable to crawl or sit down independently after 8 months. He exhibited cognitive dysfunction. After one year, he could only produce monosyllabic sounds and respond to his name. He displayed memory impairment, unable to recognize familiar objects or remember daily routines, and could neither count numbers nor perform simple calculations (see Figure 1B). Neurological examination revealed absent knee and Achilles tendon reflexes, atrophied limb muscles, and reduced muscle tone and strength. Cognitive assessment with the WISC -IV at 6.3 years indicated an IQ score of ≤40, with scores of ≤45 across all subtests, reflecting severe impairment in each domain.

**Figure 3:**
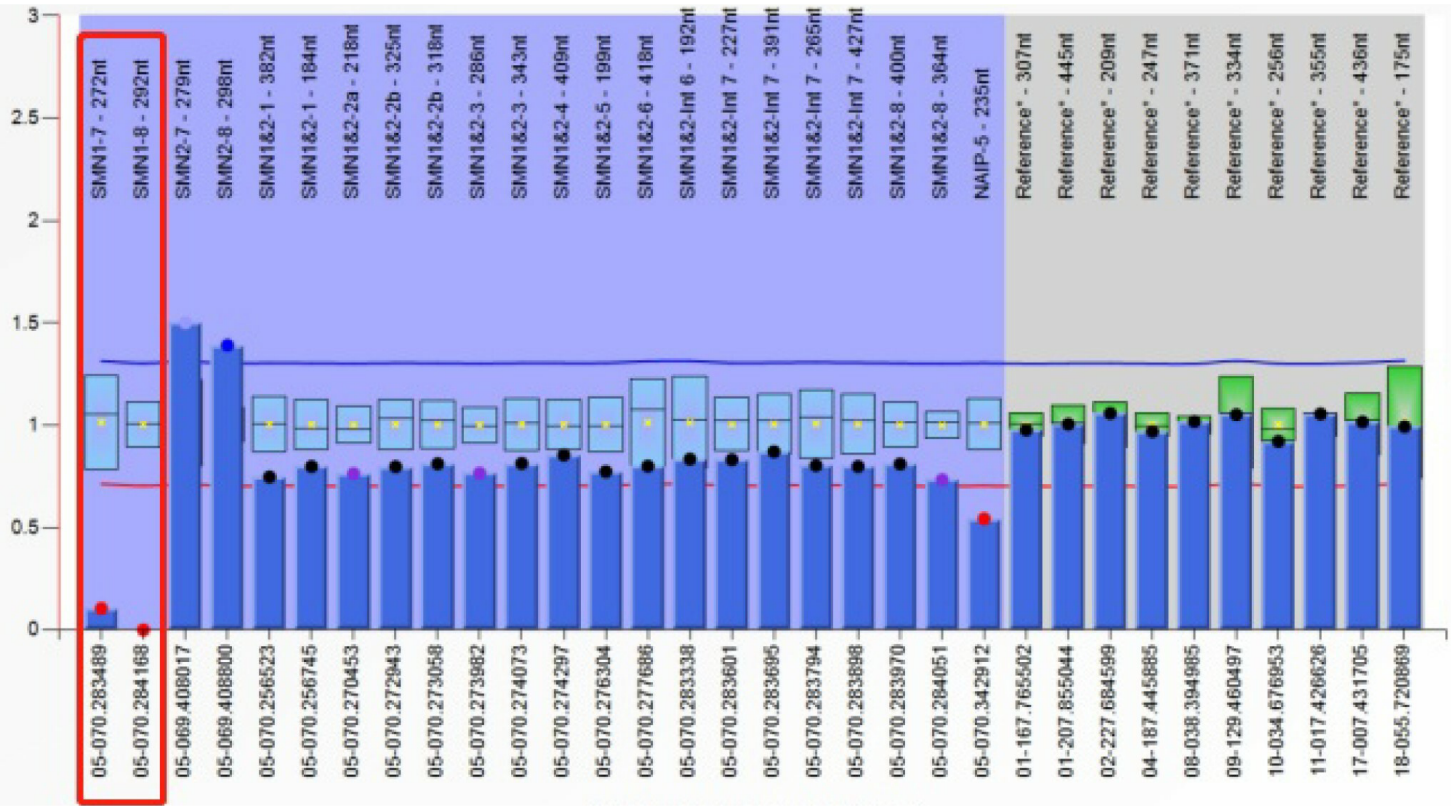
MLPA test results of patient 2.

Results from routine blood tests, blood chemistry, lactic acid, ammonia levels, and muscle enzymes were normal. Brain MRI showed scattered abnormal signals in the bilateral subcortical white matter of the frontal, parietal, and occipital lobes. The supratentorial ventricular system was slightly dilated, and the bilateral cerebral sulci were widened. Chronic denervation was noted on needle electromyography. MRI of the leg muscles revealed significant atrophy of the bilateral hip, pelvic girdle, and thigh muscles. Spinal x-ray demonstrated a scoliosis with a Cobb angle of 31 degrees. Whole exome sequencing revealed homozygous mutations in exons 7 and 8 of the SMN1 gene on chromosome 5q13. The MLPA assay indicated a copy number of 0 for exons 7 and 8 of the SMN1 gene and a copy number of 3 for exons 7 and 8 of the SMN2 gene (see Figure 3).

The onset began at 5 months with early motor milestones followed by a progressive decline. Diagnosis of 5q-spinal muscular atrophy type 1 was confirmed through genetic testing and an increased SMN2 copy number. He received four loading treatments and four maintenance treatments of nusinersen from 6.6 years of age. He received four doses of loading therapy and four doses of maintenance therapy with nusinersen. Post-treatment WISC -IV scores showed no improvement in cognitive function, and the CHOP-INTEND score also showed no significant improvement in motor function.

## Discussion

SMN2 gene is one of the important modification genes that affect the progression of SMA disease. Most SMA type 1 patients have 2 copies of SMN2, type 2 patients have 3 copies of SMN2, and type 3 patients have 3–4 copies of SMN2 (23). The number of SMN2 gene copies is negatively correlated with the severity of SMA. However, it does not fully correspond to the clinical phenotype because not all SMN2 copies are functionally identical. Intragenic SMN2 mutations, partial SMN2 deletions or duplications, and different degrees of SMN2 promoter methylation may further modify the functionality of the SMN2 gene (24, 25). Some studies that conducted sequencing analysis of the SMN2 gene found that c.859G>C mutations are associated with milder cases. Because the SMN2 genes that contain this rare variant would produce a higher number of full-length transcripts and thus of functional protein. However, the SMN2 c.859C>G variant is present in a few patients with chronic SMA but not in type 1 (26). The two patients in this study had 3 SMN2 copies, but still showed severe clinical symptoms and cognitive impairment, which may be related to these factors. Recent studies have revealed the existence of new modifier genes associated with SMA. Neuronal Apoptosis Inhibitory Protein gene (NAIP) and Small EDRK-rich factor 1A (SERF1A) are located in the 5q13.2 region. SERF1A gene can regulate the aggregation of SMN proteins. The function of NAIP gene is related to negative regulatory factors of motor neuron apoptosis. About half of patients with severe SMA lack the NAIP and SERF1A genes (27). According to previous studies, the NAIP gene is deleted in more than 50% of patients with type 1, but the frequency of this gene deletion is much lower in patients with type 2 and 3 (28). Medrano et al. (29) found in their study of SMA phenotypes in children that nearly 73% of children with type 1 lacked NAIP gene and 35% of children with type 1 lacked the SERF1A gene.

Both patients we reported had copy number of 3 copies of the SMN2 gene but were clinically typed as type 1. Detection of the patients’ SMN1 and SMN2 genes by MLPA did not reveal other SMA modifier genes. SMA patients are less likely to have genetic alterations that lead to phenotypic inconsistencies. However, further genetic testing is required to check for alterations such as mutations, partial deletions, or duplications within the SMN2 gene in these two patients.

MRI studies have revealed changes in brain white matter and brain volume in patients with SMA, which may be associated with cognitive impairment. Some studies have identified diffuse abnormal signals in the white matter of SMA patients, consistent with the MRI findings in patient 2 of this study (30). Additionally, other research has indicated that SMA patients may experience a reduction in brain volume, potentially reflecting abnormalities in neurodevelopment or neurodegenerative changes (31). These imaging findings support the notion that SMA is not only a motor system disorder but also involves alterations in cognitive function and brain structure. Therefore, a more detailed assessment and monitoring of cognitive function in SMA patients are necessary. This approach can enhance our understanding of the comprehensive impact of the disease and help optimize treatment strategies.

Although the clinical phenotype and natural history of SMA type 1 are well known in terms of motor, breathing, and swallowing functions, cognitive development in children and adolescents with this chronic disease has not received much attention. Communication has important effects on neurodevelopment, especially socialization, learning and education. Respiratory muscles and medulla oblongata muscles are the engines of speech function, and the respiratory muscles of SMA type 1 patients are seriously affected (32). Speech development is often absent or very limited in SMA type 1 patients, which significantly limits social interaction in children with SMA type 1. There are few studies on cognitive dysfunction in 5q-SMA patients. Existing studies evaluating cognitive function in SMA patients often do not specifically report or analyze those with cognitive impairment. The severely impaired motor and speech abilities in SMA children hinder accurate assessment of cognitive function, which may lead to an underestimation of their cognitive abilities (33). This is the first report of cognitive impairment in 5q-SMA patients in which two SMA type 1 patients developed cognitive impairment at an early stage.

The loss of motor ability may lead to a selective development of learning skills and cognitive abilities in SMA patients, potentially making them appear more intellectually capable compared to healthy individuals. However, SMA patients (mainly types 1 and 2) experience severe weakness that affects hand coordination and speech acquisition. The limited interaction between sensory motor skills and speech development can contribute to cognitive impairment (6). Two patients with SMA had significant cognitive impairment in our study, with severe motor limitations and lack of speech at an early stage. Children with SMA type 1 have difficulty communicating due to their inability to speak and poor motor control. Severe motor paralysis in SMA type 1 may be related to cognitive delays (11, 34). The cognitive abilities of SMA patients are related to motor dysfunction, with those experiencing greater motor difficulties showing poorer performance in attention (21). Studies on cognitive function and disease severity in SMA have found that, in patients with higher disease severity, there is lower attention and working memory ability, but better performance in verbal and verbal fluency tests (13). Motor dysfunction in SMA patients may not have a clear correlation with visual-spatial cognitive ability. A study on the cognitive and visual-spatial abilities of children with SMA type 2 found that these children did not have difficulty with complex spatial relations, and motor disorders were not a key risk factor for the significant slowdown in the development of spatial search skills (15).

The cognitive function in SMA patients may be related to the age of onset. The influence of cognitive factors may not be related to the early disease itself, the degree and duration of physical disability, but to the onset of movement disorders in early life. Children with early-onset SMA can compensate for their physical disabilities through cognitive development, which may result in higher scores in various cognitive abilities (16). As SMA patients reach adolescence, they may “compensate” for their physical deficits by acquiring cognitive skills and knowledge, with their environment facilitating higher levels of intelligence (4). The two patients described in this study were both SMA type 2 and exhibited cognitive impairment in infancy. This may be attributed to early motor disorders leading to reduced social communication and a lack of formal education, resulting in noticeable cognitive dysfunction. Family background, social factors, and access to appropriate education are likely to play a significant role in improving cognitive function in SMA patients. Without strong support and encouragement in these areas, SMA children may fail to develop compensatory mechanisms, leading to cognitive impairment (16).

Some studies suggest that physical activity in healthy teenagers positively impacts cognition. However, physical disability in early life might also positively influence cognitive function in other ways (35, 36). The severe physical disability in childhood and adolescence experienced by SMA patients often leads them to focus more on education. As a result, their cognitive function, which relies heavily on knowledge and education, may improve compensatorily. Severe dyskinesia may lead to educational disadvantages, but early educational support appears to stimulate compensatory development (16).

The two patients in our study are SMA type 1, with early onset and noticeable cognitive impairment in infancy. They did not receive formal education after birth. The gradual lack of stimulation and limited social experiences in older children with SMA type 1 may contribute to a gap between cognitive ability and language comprehension. A study on task performance in SMA type 1 patients found that these patients had poorer task completion. Although children with SMA type 1 may attend school regularly and receive formal education, they might still experience difficulties in remembering, processing, or expressing cognitive information. Additionally, children with SMA type 1 may start school later and experience more comorbidities and absences, which can affect their learning and contribute to cognitive impairments (7). SMA primarily affects motor function and usually has minimal impact on cognitive function. Few studies have shown that SMA can impact cognitive function, though it typically does not result in severe cognitive impairment. Cognitive dysfunction may be a co-morbidity in these two SMA patients, and the underlying causes of their severe cognitive impairment require further investigation.

There is little evidence in studies about cognitive performance in children with SMA, but children with SMA type 1 are more likely to be affected. Even children who are cognitively capable at birth may experience cognitive delays due to a lack of cognitive stimulation (7). Current research suggests that cognitive outcomes may be related to the copy number of the SMN2 gene. The deficiency of SMN protein not only impacts spinal motor neurons but may also affect other cellular components of the central nervous system. Severe reductions in SMN protein levels may lead to progressive brain dysfunction and degeneration. Some cases of SMA without SMN2 (SMA type 0) exhibit atrophy of the white matter and hippocampus, with high signals observed in the thalamus and basal ganglia on magnetic resonance imaging (8). Wishart et al. (37) demonstrated that alterations in brain development processes were associated with low SMN protein levels in severe SMA mouse models. Studies on brain structure changes in adults with SMA and healthy controls found cerebellar atrophy and increased gray matter density in the motor cortex. Cortical hypertrophy in motor areas has been interpreted as a form of cortical reorganization following lower motor neuron degeneration (38). It remains unclear whether SMN2 deficiency in the central nervous system is the primary cause of reduced intelligence quotient (IQ), or if muscle weakness limits patients’ ability to explore their surroundings, thereby hindering IQ development (19). The abnormal cranial MRI findings in patient 2 suggest that atypical brain development in SMA patients may be linked to cognitive dysfunction.

## Conclusion

Cognitive impairment may occur in SMA patients, but relevant studies are limited. Cognitive impairment in SMA patients might be associated with motor impairment, age of onset, and education, rather than the underlying gene mutation. The pathogenesis may involve brain developmental disorders and SMN protein deficiency. SMA patients typically exhibit mild cognitive changes, but this study reports two SMA type 1 patients with severe cognitive dysfunction, which may be a co-morbidity of SMA. Clinicians should be cautious not to underdiagnose SMA or misdiagnose it as another condition, especially in patients with muscle weakness and atrophy. Early cognitive intervention is recommended to prevent progressive cognitive impairment in SMA patients. The relationship between cognitive dysfunction and SMA is under-researched, and there is no evidence linking cognitive characteristics with nusinersen treatment. Further research and exploration are needed in these areas in the future.

## Data Availability

The original contributions presented in the study are included in the article/Supplementary Material, further inquiries can be directed to the corresponding author.
